# Direct Interaction between Two Viral Proteins, the Nonstructural Protein 2C^ATPase^ and the Capsid Protein VP3, Is Required for Enterovirus Morphogenesis

**DOI:** 10.1371/journal.ppat.1001066

**Published:** 2010-08-26

**Authors:** Ying Liu, Chunling Wang, Steffen Mueller, Aniko V. Paul, Eckard Wimmer, Ping Jiang

**Affiliations:** Department of Molecular Genetics and Microbiology, School of Medicine, Stony Brook University, Stony Brook, New York, United States of America; University of California San Francisco, United States of America

## Abstract

In spite of decades-long studies, the mechanism of morphogenesis of plus-stranded RNA viruses belonging to the genus *Enterovirus* of *Picornaviridae*, including poliovirus (PV), is not understood. Numerous attempts to identify an RNA encapsidation signal have failed. Genetic studies, however, have implicated a role of the non-structural protein 2C^ATPase^ in the formation of poliovirus particles. Here we report a novel mechanism in which protein-protein interaction is sufficient to explain the specificity in PV encapsidation. Making use of a novel “reporter virus”, we show that a quasi-infectious chimera consisting of the capsid precursor of C-cluster coxsackie virus 20 (C-CAV20) and the nonstructural proteins of the closely related PV translated and replicated its genome with wild type kinetics, whereas encapsidation was blocked. On blind passages, encapsidation of the chimera was rescued by a single mutation either in capsid protein VP3 of CAV20 or in 2C^ATPase^ of PV. Whereas each of the single-mutation variants expressed severe proliferation phenotypes, engineering both mutations into the chimera yielded a virus encapsidating with wild type kinetics. Biochemical analyses provided strong evidence for a direct interaction between 2C^ATPase^ and VP3 of PV and CAV20. Chimeras of other C-CAVs (CAV20/CAV21 or CAV18/CAV20) were blocked in encapsidation (no virus after blind passages) but could be rescued if the capsid and 2C^ATPase^ coding regions originated from the same virus. Our novel mechanism explains the specificity of encapsidation without apparent involvement of an RNA signal by considering that (i) genome replication is known to be stringently linked to translation, (ii) morphogenesis is known to be stringently linked to genome replication, (iii) newly synthesized 2C^ATPase^ is an essential component of the replication complex, and (iv) 2C^ATPase^ has specific affinity to capsid protein(s). These conditions lead to morphogenesis at the site where newly synthesized genomes emerge from the replication complex.

## Author Summary

Enteroviruses are single, plus-stranded RNA viruses that contain a large number of closely related pathogens. Human enteroviruses cause altogether >3 billion human infections per year, inflicting diseases ranging from benign (common cold) to very serious (poliomyelitis). Enterovirus replication has been studied for decades yet the mechanism of genome selection during encapsidation, a key step open for chemotherapeutic intervention, remains unknown. Attempts to identify a genomic “packaging signal”, instructing the genome to engage with capsid proteins in morphogenesis, have failed. We have used the similarities and dissimilarities of two closely related subspecies of enteroviruses, poliovirus and coxsackie A viruses (CAVs), and constructed chimeras in which the capsid coding region was interchanged. A chimera, with the CAV capsid domain and the poliovirus two non-structural domains of the polyprotein, synthesized its genome with wt kinetics yet was blocked in morphogenesis. Genetic and biochemical studies of chimeras led to the discovery that the non-structural protein 2C^ATPase^ (essential part of the replication complex) and capsid protein VP3 must directly interact for morphogenesis to proceed. This has led us to propose a novel mechanism by which the specificity of enterovirus morphogenesis is governed by protein-protein rather than RNA-protein interaction at the site of genome synthesis.

## Introduction

Morphogenesis is a crucial step at the end of the virus' life cycle that provides newly synthesized genomes with a protective shell to survive in the extracellular environment yet assures attachment to and penetration into subsequent host cells. Morphogenesis of viral genomes must be specific because encapsidation of non-progeny nucleic acid is wasteful for the virus, for which reason elaborate mechanisms have evolved to discriminate against nucleic acids other than its own genome.

Here we describe our studies of the morphogenesis of a group of single, plus-stranded RNA viruses that belong to the genus *Enterovirus* of *Picornaviridae*, a family of viruses containing a large number of human and animal pathogens. Poliovirus (PV), the prototype enterovirus, has been extensively studied for a century and although much is known about its virion structure, uptake into host cell, genome structure and macromolecular events of replication, the mechanism of particle assembly is only poorly understood [1]. The key requirement of morphogenesis, namely the specific selection of viral genomes, has also remained obscure. We have discovered a novel mechanism for enteroviruses in which the specificity of encapsidation is facilitated by protein-protein interaction. It should be noted that this mechanism is different from the one used by some other RNA viruses such as hepatitis B virus and alphaviruses [2], [3]. The specificity of encapsidation with these viruses is dependent on an RNA encapsidation signal and RNA/protein interactions.

**Figure 1:**
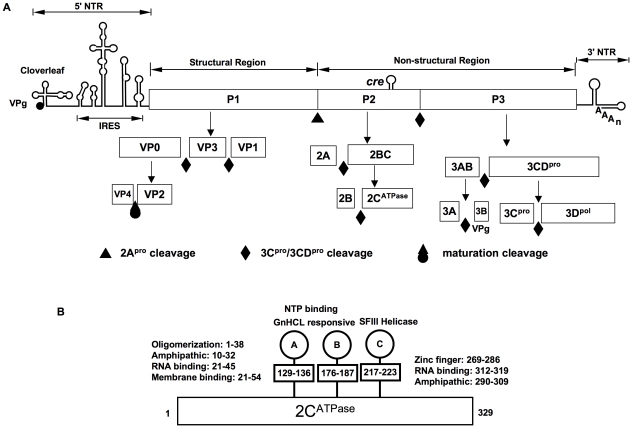
The genomic organization of C-HEV RNAs, proteolytic processing of the polyprotein and PV 2C^ATPase^ motifs. (**A**) The single-stranded genomic RNA is covalently linked to the viral-encoded protein VPg at the 5′ end of the NTR (5′NTR). The 5′NTR consists of the cloverleaf, and the IRES. The coding region (open box) depicts the structural (P1) and nonstructural (P2 and P3) regions. Within the P2 (2C^ATPase^) coding region, the *cis* replication element (*cre*) is indicated. The 3′NTR contains a heteropolymeric region and is polyadenylated. The precursors and mature cleavage products of the P1, P2 and P3 domains along with their cleavage sites are shown. (B) Functional motifs in 2C^ATPase^. Motifs A and B are homologous to those in NTP binding/hydrolyzing proteins, motif C shares homology to superfamily helicase III. Two RNA binding motifs, two amphipathic helixes, the N-terminal membrane binding motif, oligomerization motif and the C-terminal zinc-binding motif are indicated. Amino acid positions of each motif are numbered.

Enteroviruses synthesize only one protein, the polyprotein, which is cleaved by two virus-encoded proteinases, 2A^pro^ and 3C^pro^/3CD^pro^, into intermediates expressing specific functions (e.g. 3CD^pro^) and into mature proteins (Figure 1A). After its release from the polyprotein by 2A^pro^, the precursor of the structural proteins (P1) interacts with cellular chaperone Hsp90 [4], a requirement for its subsequent processing by 3CD^pro^ into capsid proteins VP0, VP3 and VP1 (Fig. 1A) [5]. These cleavage products will spontaneously form a 5S protomer (VP0, VP3, VP1) that can oligomerize to the 14S pentamer (VP0, VP3, VP1)_5_; twelve pentamers, subsequently, assemble into a 75S empty capsid [(VP0, VP3, VP1)_5_]_12_, also called procapsid [6], [7]. It is not known at what stage progeny genomes interact with the capsid precursors. They may be inserted into the procapsid or, alternatively, pentamers may condense around RNA emerging from the replication complex. Either process will yield provirions {[(VP0, VP3, VP1)_5_]_12_RNA} [8], [9], [10] that mature to virions when VP0 is cleaved to VP4 and VP2 by a mechanism possibly involving an RNA-dependent autocatalytic process [6], [7].

The encapsidation process in PV morphogenesis is highly restricted to newly synthesized plus strand progeny RNA [11], [12]. Under normal conditions of replication in HeLa cells, cellular RNAs, PV mRNA lacking VPg or viral VPg-linked minus strand RNA are excluded from mature viral particles [6]. Numerous studies aimed at determining the specificity of encapsidation by searching for an RNA packaging signal have been unsuccessful. The very long 5′NTR of PV can be replaced with that of the distantly related coxsackie B3 virus (CVB3) [13] or CVB4 [14] yielding virions containing chimeric genomes that proliferate with PV wild type (wt) kinetics. Similarly, the cloverleaf of PV can be changed to that of HRV2 [15], or the PV IRES has been exchanged with IRESes from other picornaviruses [16], [17], [18] and even with that of HCV [19] without yielding impaired encapsidation phenotypes. The 3′NTR of PV, in turn, has been exchanged with that of HRV14, a single stem-loop structure with no apparent similarity in structure and sequence to that of PV. This chimera too proliferated with wt kinetics [20]. This makes it highly unlikely that the 5′- and 3′-NTRs of poliovirus contain packaging determinants. The genomic sequence encoding the capsid P1 precursor cannot harbor an encapsidation signal since the entire P1 encoding region can be deleted [21] or replaced by foreign genes [14], [22], [23]. Such PV replicons, all of which can replicate, can be efficiently encapsidated with PV capsid proteins in *trans*. Recent experiments from this laboratory, employing a “scrambled” sequence [24] of the P2 coding region (scramble of synonymous codons) have eliminated this region too from carrying an encapsidation signal (Song, Mueller, Ward, Skiena, Futcher, Paul and Wimmer, manuscript in preparation). Finally, genetic modification of the PV VPg coding sequence [25], [26] or engineering PVs carrying VPg sequences of other picornaviruses [25], [27], [28] have also eliminated the VPg coding sequence as providing an encapsidation signal. VPg, however, may still play a role in encapsidation (see below). Currently it seems unlikely that poliovirus or other enteroviruses (including the rhinoviruses that have recently been classified as enteroviruses) harbor an RNA signal that would instruct the capsid components to bind to and enclose the viral genome in a specific manner. Only one member of the extended family of *Picornaviridae,* Aichi virus (*Kobuvirus* genus), was reported to contain a 5′-terminal RNA stem loop with a role in particle assembly [29].

Among the nonstructural proteins of PV, 2C^ATPase^ and 3CD^pro^, have been reported to be involved in packaging although no mechanism(s) is known. Studies with an *in vitro* translation/RNA replication system, which produces viable viruses [11], have suggested that 3CD^pro^ functions at a late step in the assembly process just before or during the maturation cleavage of VP0 to VP2 and VP4 [30]. Protein 2C^ATPase^ of PV has been implicated in virion capsid formation through genetic analysis of a cold-sensitive mutant [31] or by determining escape mutants from drug (hydantoin) inhibition [32]. The multifunctional 329 amino acids-long 2C^ATPase^ is the most complex and least understood nonstructural proteins of enteroviruses. The functions of this protein that are highly conserved among picornaviruses, include, in addition to encapsidation, host cell membrane rearrangements [33], [34], genome replication [35], [36] and even uncoating of viral particles [37]. Based on sequence analyses the protein has been classified as a member of the superfamily III helicases, which contain 3 conserved motifs (A–C), including two classical ATP binding motifs (A and B) (Fig. 1B) [38]. Purified 2C^ATPase^ possesses ATPase activity [39], [40], which is inhibited by guanidine HCl (GnHCl) [41], a known potent inhibitor of PV RNA replication [18]. *In vitro* the protein forms homo-oligomeric structures required for ATPase activity [42]. The N-terminal part of the protein contains a RNA binding domain and an amphipathic helix, which is involved in membrane binding and oligomerization [36], [42], [43]. Another amphipathic helix, a RNA binding domain and a cysteine rich domain that binds zinc are located near the C-terminus [44], [45]. In infected cells 2C^ATPase^ appears to be associated with viral RNA in the replication complexes on the surface of membranous vesicles [46].

Available evidence suggests that genome replication is a precondition of PV encapsidation [11], [12]. Electron-microscopic studies, which showed that RNA replication complexes co-localize with capsid precursors on membranous vesicles during infection [47], supported these observations. Nugent et al. [12] hypothesized that encapsidation specificity may be determined by the spatial arrangement of replication complexes with the capsid precursors. This intriguing hypothesis lacked an essential component: what brings the capsid precursors into the vicinity of the replication complexes since PV replicons lacking the P1 domain altogether can be efficiently encapsidated in *trans*?

Human enteroviruses have been divided into several clusters based on genotype relationships [48]. PV types 1–3, and eleven C-cluster coxsackie A virus serotypes share the C-cluster, also referred to as C-cluster human enteroviruses (C-HEVs). Their difference in affinity to cellular receptors, PVs using CD155 [49], [50] while C-CAVs using ICAM-1 [51], accounts for significant capsid dissimilarities between the member viruses of this species [52], [53]. In contrast, the differences between the non-structural proteins of PV vs C-CAVs are less pronounced. We have used the similarities and dissimilarities between PV and C-CAVs to separate RNA replication from encapsidation by constructing chimeric viruses in which capsid precursor P1 and/or 2C^ATPase^ have been exchanged. All of the chimeric viruses studied here replicated their genomes with wt kinetics in tissue culture cells but were blocked in encapsidation, a phenotype that we examined by genetic analyses. We present genetic evidence suggesting a specific interaction between 2C^ATPase^ and VP3, which is essential for genome encapsidation. The genetic evidence of the 2C^ATPase^-VP3 interaction was further substantiated by biochemical assays. We propose that the primary determinant of encapsidation specificity in the enterovirus life cycle is protein-protein interaction.

## Materials and Methods

### Cells

HeLa H1 cells were maintained in DMEM (Life Technology), supplemented with 10% FCS, 100 units of penicillin, and 100 mg of streptomycin per milliliter.

### Viruses

The prototype strain of C-CAVs, CAV20, CAV21 (Kuykendall) and CAV18, propagated in HeLa H1 cells, were obtained from the American Type Culture Collection. Polioviruses (PVM) were derived from cDNA pT7PVM [54] by transfection.

### Plasmids

*Parental plasmids of PV:* pT7PVM contain a full-length infectious cDNA of PVM.

*Parental plasmids of C-CAVs:* pT7CAV20 contains a full-length infectious cDNA of CAV20 [48]. pGEM-CAV21 and pT7CAV18, which contain a full length infectious cDNA of CAV21 and CAV18 (Kuykendall), respectively, were constructed by Elizabeth Rieder.

*Chimeric genomes:* Parental plasmids of PVs and C-CAVs were used as the backbone for cloning as described below. All plasmids contain the T7 promoter in front of the 5′ end of the full length genomic cDNA for *in vitro* RNA transcription by T7 RNA polymerase [54].

### Construction of chimeric genomes between C-CAVs

Parental plasmid cDNAs of CAV20, CAV21 and CAV18 were used as the backbone for cloning. Using a three-step overlapping PCR, chimeras between CAV20 and CAV21 (or CAV18) were generated by precise swapping of the genetic segment encoding the P1 region of the polyprotein [48]. The oligonucleotides and the templates for PCR are summarized in Supplementary Table 2 in Text S1. The overlapping PCR fragment and the vectors were digested with the same pair of restriction enzymes and ligated to produce the cDNA clone of the chimeric genome. The vectors and the restriction sites for construction of the chimeric genomes are listed in Supplementary Table 3 in Text S1. The DNA sequence of the final constructs was verified by sequencing analysis using BigDye Kit and ABI Prism DNA sequencer (model 310). Replacement of the original 2C^ATPase^ coding region in each of the chimeric genome with that of the same origin as the capsid coding region follows the same strategy described above (Supplementary Table 2 & 4 in Text S1).

### Construction of C_20_PP derivatives

Construction of the chimeric C_20_PP genome was described before [48]. For construction of the C_20_PP derivatives, listed in Supplementary Table 3 in Text S1 and Figure 4, a two-step overlapping PCR similar to the one described above was performed using C_20_PP as the template with the mutation(s) introduced into the internal primers (Supplementary Table 3 & 4 in Text S1).

### Construction of Renilla luciferase reporter viruses

To test the RNA replication efficiency of chimeric viruses, we used novel reporter viruses, which contain the Renilla luciferase gene fused to the N-terminal of the P1 coding region of the chimeric genomes. The same strategy (three-step overlapping PCR), described above, was used to introduce the Renilla luciferase gene into the N-terminal of P1 the coding region of the chimeric genomes (Supplementary Table 3 & 4 in Text S1). The luciferase protein is post-translationally cleaved from the remainder of the polyprotein by 3CD^pro^ at a recombinant 3CD^pro^ cleavage site.

### Analysis of the growth phenotypes of the parental and chimeric viruses

Parental plasmid cDNAs pT7PVM, pT7CAV20, pGEM-CAV21, pT7CAV18 and the chimeric constructs were linearized at a unique restriction sites downstream the poly(A) tract (Supplementary Table 5 in Text S1) and used as templates for *in vitro* RNA synthesis using T7 RNA polymerase. RNA transcripts were transfected into HeLa H1 cell monolayers by the DEAE-Dextran method as described before [54]. Following transfection, virus was harvested from the transfected cells when 90–95% of the cells displayed cytopathic effect (CPE). Lysates of transfected cells from the chimeric genomes showing no CPE were inoculated into 35-mm-diameter HeLa H1 cell monolayers for 6–8 subsequent serial passages. The plaque phenotypes and virus titers (PFU/ml) of the parental and chimeric viruses were determined in triplicate by plaque assay [11] using 0.6% tragacanth gum. The identity of the chimeric viruses was confirmed by RT-PCR/sequencing analysis.

### *In vitro* translation

*In vitro* RNA translations were performed with HeLa cell S10 cytoplasmic extracts at 34 degree Celsius as described previously [11].

### RT-PCR and sequencing analysis of viral RNA

HeLa H1 cell monolayers (5×10^6^) were infected with viruses that were purified from plaque assay. At 7-hr post infection, total cytoplasmic RNA was extracted with 1 ml Trizol reagent (Invitrogen) and amplified into DNA using Titan one tube RT-PCR system (Roche). The RT-PCR products were sequenced using the Bigdye Terminator Sequencing Kit (ABI, Applied Biosystems).

### Luciferase assay

Dishes (35-mm diameter) of monolayered HeLa H1 cells were transfected with 5 µg of replicon RNAs and were incubated at 37 degree Celsius in standard tissue culture medium in the presence and absence of 2 mM GnHCl. Luciferase activities were determined in lysates of cells harvested 16 hrs after transfection. Cell lysates (10 µl) was mixed with 20 µl of luciferase assay reagent (Promega; luciferase assay system catalog no. E2810) and Renilla luciferase activity was measured in an Optocomp I luminometer (MGM Instruments, Inc.). Cell lysates from transfections were used to re-infect HeLa H1 cells in the presence and absence of 2 mM GnHCl and luciferase activities were determined in lysates of cells harvested 8 hrs after infection. Luciferase activity ratio (−GnHCL/+GnHCl) represents: luciferase activity without GnHCl divided by luciferase activity with GnHCl in either transfection or infection.

### Construction of His-tagged VP3

A PCR fragment containing full length PV VP3 was amplified and cloned into the pET21b vector (Novagen) with the restriction enzymes Sac I and Xho I.

### Purification of GST-2C^ATPase^ and His-tagged VP3 proteins

GST-tagged 2C^ATPase^ and His-tagged VP3 recombinant proteins were expressed in *E. coli*. The GST-2C^ATPase^ proteins were expressed from pGEX-2C vector and purified by glutathione sepharose column (GE Healthcare) as described before [41]. The His-VP3 proteins were purified by nickel column chromatography (QIAGEN).

### GST pull-down assay

Briefly, 5 µg GST-2C^ATPase^ (or 2 µg GST as a control) were incubated with glutathione sepharose beads at 4°C for 3 hr in buffer containing 50 mM Tris-HCl pH7.5, 140 mM NaCl, 0.1% TritonX-100 with protease inhibitor cocktail tablets (Roche). The protein bound GSH beads were washed with PBS 3 times and then 5 µg His-VP3 was added. After 1 hr incubation at 4 degree Celsium, the glutathione beads were washed 3 times and were boiled in 1x SDS sample buffer for 5 min. The samples were analyzed by SDS-polyacrylamide gel (12.5% acrylamide) electrophoresis and followed by western blot analysis using antibodies against PV VP3 (polyclonal, kindly contributed by Dr. Delpeyroux, Pasteur Institute, France).

### Co-immunoprecipitation assay using 2C^ATPase^ and VP3 proteins translated in an *in vitro* cell-free translation system

Plasmids used for *in vitro* translation of CAV20 structural protein VP3 (wt), VP3 (E180G) and CAV20 non-structural protein 2C^ATPase^ (wt), PV non-structural proteins 2C^ATPase^ (wt) and 2C^ATPase^ (N252S) were generated with A2 plasmid [55] and PCR fragments encoding wt and mutant proteins of VP3 and 2C^ATPase^ according to methods described previously [55]. 2 µg of each VP3 and 2C RNA transcripts generated *in vitro* by T7 RNA polymerase were co-translated in HeLa extract and labeled by ^35^S-labeled methionine. Using anti-PV 2C polyclonal antibody, Co-IP assay was performed with co-translated ^35^S labeled 2C^ATPase^ and VP3 proteins following standard protocols using protein A/G plus-agarose (Santa Cruz Biotechnology) and [56]. The radioactive signals from input proteins and Co-IP reaction products were quantified by a PhosphorImager (Molecular Dynamics, Storm 860) by measuring the amount of ^35^S incorporated into product. Interactions between 2C^ATPase^ and VP3 were represented by percentages of the levels observed in the Co-IP reaction with CAV 2C^ATPase^ and CAV VP3 after normalizing the amount of input 2C^ATPase^ and VP3. Numbers given for the extents of interactions represented the average of three independent experiments.

## Results

### Rationale for generating PV and CAV chimeras to study enterovirus encapsidation

**Figure 2:**
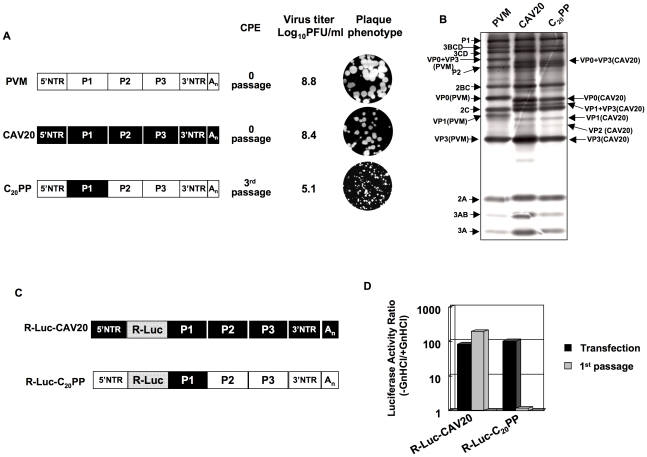
Chimeric virus C_20_PP containing the capsid-coding region of CAV20 is defective in encapsidation. (**A**) Growth phenotypes of the parental and of chimeric viruses. The genomic structures of the parental and of chimeric viruses used are illustrated on the left. PVM sequences are shown with open boxes, CAV sequences are shown by black boxes. Nomenclature used for the chimeric viruses: the first letter refers to the origin of the P1 region, the 2nd and 3rd letters refer to the origins of the P2 and P3 regions, respectively. RNA transcripts were transfected into HeLa H1 cells and the viruses obtained at CPE, if any, were titered by a plaque assay (Materials and Methods). (**B**) C_20_PP is normal in translation and polyprotein processing. RNA transcripts (500 ng) derived from wt PVM, wt CAV20 and C_20_PP were translated *in vitro* in HeLa cell extracts at 34 degree Celsius ° overnight, as described in Material and Methods. The reaction mixtures were analyzed by SDS/PAGE with 12% acrylamide and the viral proteins were visualized by autoradiography. The positions of the precursor and mature proteins after processing are indicated. (**C**) The genomic structures of the parental and of chimeric luciferase reporter viruses used are illustrated. The R-Luc gene is shown in dotted box. (**D**) Comparison of RNA replication and encapsidation of the parental and of chimeric reporter viruses. To determine the level of RNA replication (black bar), RNA transcripts were transfected into HeLa H1 cells both in the absence and presence of 2 mM GnHCl. To determine encapsidation (grey bar), the transfected cell lysates were passaged into fresh HeLa H1 cells in the absence and presence of GnHCl. The luciferase activity in the absence (−GnHCl) and presence of 2 mM GnHCl (+GnHCl) was measured and the ratio of luciferase activity (–GnHCl/+GnHCl) was calculated to quantify the extent of replication (Materials and Methods). The luciferase data are the averages of at least three independent experiments.

The lack of evidence for an RNA packaging signal in enterovirus proliferation has prompted us to study the specificity of encapsidation by searching for possible protein-protein interactions needed for this process. Previous studies have shown that chimeric constructs of the PV polyprotein with exchanges of varying coding regions of closely related picornaviruses can be utilized to analyze determinants of viral macromolecular interactions and replication [57], [58], [59], [60], [61], [62]. PV and C-CAVs share a high degree of amino acid identity in their nonstructural proteins but are not closely related in their capsid sequences probably because they evolved to use different cellular receptors [48]. In a chimera with the capsid of one C-HEV and the nonstructural proteins of another C-HEV, this difference may produce specific morphogenesis phenotypes due to incompatibility or poor interaction between capsid and nonstructural proteins. In our previous work, we have already observed that the replacement of the PV type 1 Mahoney (PVM) capsid with that of its closest relative, CAV20, resulted in a quasi-infectious virus, C_20_PP (Figure 2A) [48]. In C_20_PP, the first letter refers to the origin of the P1 region, the 2nd and 3rd letters refer to the origins of the P2 and P3 regions, respectively. The quasi-infectious phenotype means that a step in the life cycle of C_20_PP is so severely debilitated that only escape variants can be recovered from transfections with RNA transcripts [48]. The molecular basis of the defective phenotype of C_20_PP was not elucidated but could be proteolytic processing of the capsid precursor, genome replication or encapsidation. The observed quasi-infectious phenotype of C_20_PP made it possible to subject this chimera to a genetic analysis that may reveal a defect in encapsidation.

### C_20_PP is defective in encapsidation

We first provided evidence that the quasi-infectious phenotype of C_20_PP was not due to abnormal translation or protein processing resulting from poor compatibility between the heterologous capsid and the 3CD^pro^ polypeptide in C_20_PP. Translation of RNA transcripts of wt and chimeric constructs in a HeLa cell-free extract [11] showed normal translation and protein processing patterns (Figure 2B).

The search for the block of C_20_PP proliferation led us to develop a novel reporter virus in which the PV open reading frame (ORF) of the Renilla Luciferase (R-Luc) protein was fused to the N-terminus of the viral polyprotein. In the course of the infection the R-Luc is cleaved off from the viral polyprotein at an engineered 3CD^pro^ proteinase cleavage site. Due to the small size of the inserted R-Luc gene this virus was stable for 1 passage after transfection and, thus, suitable for our experiments. This construct is similar to a previously described recombinant coxsackie B3 virus that stably expressed eGFP in tissue culture [63]. The advantage of using our reporter virus over conventional reporter replicons, in which the P1-coding sequence is replaced by the luciferase gene, is that it can distinguish between a defect in replication and encapsidation. A reporter viral genome (with the R-Luc sequence) that is unable to encapsidate itself will exhibit normal RNA replication levels as evidenced by a wt-like Renilla luciferase signal after RNA transfection. However, it would not generate infectious progeny and, consequently, passage to fresh cells will fail, leading to the loss of the luciferase signal.

We have made such reporter viruses from both the parental wt CAV20 and the chimeric C_20_PP (Figure 2C). RNA transcripts were transfected into HeLa H1 cells both in the absence and presence of 2 mM GnHCl, a potent inhibitor of PV [18] and CAV20 RNA replication [18], [48]. Luciferase activity was determined at 16-hr post transfection either in the presence of GnHCl (+GnHCl) throughout the incubation period that allows us to measure the translation of the transfecting RNA, or in the absence of GnHCl (−GnHCl) when the luciferase signal is increased because of RNA synthesis. The ratio of the luciferase signals –GnHCl/+GnHCl indicates the extent of genome replication. As shown in Figure 2D there was a 100 fold increase of the luciferase signal at −GnHCl compared to +GnHCl with both wt R-Luc-CAV20 and R-Luc-C_20_PP viruses, an observation indicating robust RNA synthesis under these conditions. Lysates of transfected cells were then inoculated to fresh HeLa H1 cells as 1^st^ passage either in the presence or absence of GnHCl. Eight hours post infection the luciferase activity was measured (Figure 2D). The high level of luciferase activity obtained after the first passage of the R-Luc-CAV20 virus indicated the formation of virions that had encapsidated the wt genome in the course of transfection. In contrast, no luciferase signal could be detected after passage of the lysate harboring the R-Luc-C_20_PP chimera (Figure 2D). We conclude that the genome of the C_20_PP chimera, although competent in RNA replication, cannot form infectious progeny, e.g. it is defective in genome encapsidation. The reason for the defect in encapsidation, however, remains elusive.

### The encapsidation defect of chimera C_20_PP is rescued either by a mutation in capsid protein VP3 or by a mutation in the nonstructural protein 2C^ATPase^

**Figure 3:**
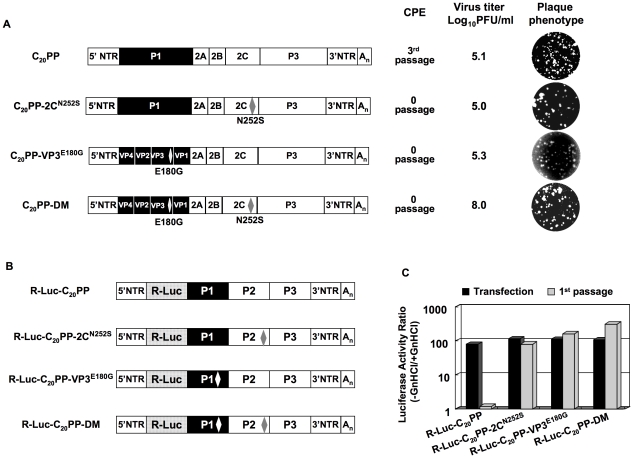
Rescue of the C_20_PP virus by mutations in 2C^ATPase^ or/and in VP3. (**A**) Growth phenotypes of the C_20_PP chimera and of its derivatives. The genomic structures of the C_20_PP virus and of its derivatives are illustrated on the left. For the nomenclature of the chimera see the legend to Figure 2A. CAV20 sequences are shown with black boxes, PVM sequences with open boxes. The amino acid changes in 2C^ATPase^ and in VP3 are shown in superscript. C_20_PP-DM represents the double mutant. RNA transcripts were transfected into HeLa H1 cells and the viruses present after complete CPE were titered by a plaque assay (Materials and Methods). (**B**) The genomic structures of the C_20_PP reporter chimeras, carrying the R-Luc gene fused N-terminal to P1, and of its derivatives. (**C**) Comparison of RNA replication and encapsidation of the C_20_PP virus and of its derivative reporter viruses. To determine the level of RNA replication (black bar), RNA transcripts were transfected into HeLa H1 cells both in the absence and presence of 2 mM GnHCl. To determine encapsidation (grey bar), the transfected cell lysates were passaged into fresh HeLa H1 cells in the absence and presence of GnHCl. Luciferase activity in the absence (−GnHCl) and presence of 2 mM GnHCl (+GnHCl) was measured and the ratio of luciferase activity (–GnHCl/+GnHCl) was calculated to quantify the extent of replication (Materials and Methods). The luciferase data are the averages of at least three independent experiments.

As mentioned above, the C_20_PP chimera is quasi-infectious. Variants that escaped the block in encapsidation were found only after three blind passages following transfection. This indicated the emergence of mutation(s). To identify the rescuing mutation(s), we plaque purified two viruses that had emerged after three passages from two independent transfections with C_20_PP transcripts. RT-PCR and sequence analyses of the viral genomes revealed two independent single mutations that mapped to the coding region of either VP3 (E180G) or 2C^ATPase^ (N252S). By separately engineering these two mutations back into the cDNA of C_20_PP we obtained two viable viruses C_20_PP-VP3^E180G^ and C_20_PP-2C^N252S^ (Figure 3A). Their titers after transfection were as low as those observed with isolates after three passage of C_20_PP (Figure 2A) and 1000 fold lower than that observed with wt CAV20. Moreover, C_20_PP-VP3^E180G^ and C_20_PP-2C^N252S^ expressed a small plaque phenotype compared to that of wt CAV20 (Figures 2A and 3A).

### The double mutation (VP3^E180G^&2C^N252S^) fully rescues C_20_PP to a wt encapsidation phenotype

The phenotypes of C_20_PP-VP3^E180G^ and C_20_PP-2C^N252S^ did not change after further passages, e.g. all attempts to isolate variants with improved proliferation phenotypes failed (data not shown). This prompted us to engineer both the VP3^E180G^ and 2C^N252S^ mutations into C_20_PP (C_20_PP-DM). Variant C_20_PP-DM expressed phenotypes (virus titer and plaque size) almost the same as that of wt CAV20 (compare Figures 2A and 3A). Currently, we cannot explain why in our experiments the C_20_PP-DM-like variant did not evolve during passaging of C_20_PP.

To confirm the rescue of the encapsidation defect of C_20_PP by the mutations in VP3 and 2C^ATPase^, we constructed reporter viruses R-Luc-C_20_PP-VP3^E180G^, R-Luc-C_20_PP-2C^N252S^ and R-Luc-C_20_PP-DM (Figure 3B). All three produced strong luciferase signals after transfection of their genomic RNAs into HeLa H1 cells, as expected (Figure 3C). After a passage into fresh HeLa H1 cells, the luciferase activity was highly impaired with C_20_PP but was found to be partially rescued if the virions carried the single 2C^N252S^ or VP3^E180G^ mutation or fully rescued by the double mutation (Figure 3C). Thus, although a single mutation can partially rescue the proliferation phenotype of C_20_PP, the double mutation 2C^N252S^/VP3^E180G^ in the genome of the chimera is capable of producing a proliferation phenotype similar to that of wt CAV20. This observation, confirms the previous genetic data by Vance et al. [32] that the coding region of 2C^ATPase^ is linked to encapsidation. More importantly, the cooperative activity of VP3 with 2C^ATPase^ suggests that capsid protein VP3 functions through a direct interaction with 2C^ATPase^ in encapsidation. As indicated before, the coding sequence of 2C^ATPase^, however, can be eliminated as carrying an encapsidation signal. The *cre*, the only known essential RNA structure in coding sequence of 2C^ATPase^, are highly homologous in sequence between PV and CAV20. Moreover, scrambling of the P2 RNA sequence has no influence on PV proliferation if the essential *cre* is transplanted to the 5′NTR (Song, Mueller, Ward, Skiena, Futcher, Paul, and Wimmer, manuscript in preparation). It is, thus, likely that VP3 and 2C^ATPase^ or their precursors cooperate by protein-protein interaction, a novel mechanism for specific genome encapsidation of enteroviruses.

### A CAV20-like 2C^ATPase^ rescues the encapsidation defect of C_20_PP

**Figure 4:**
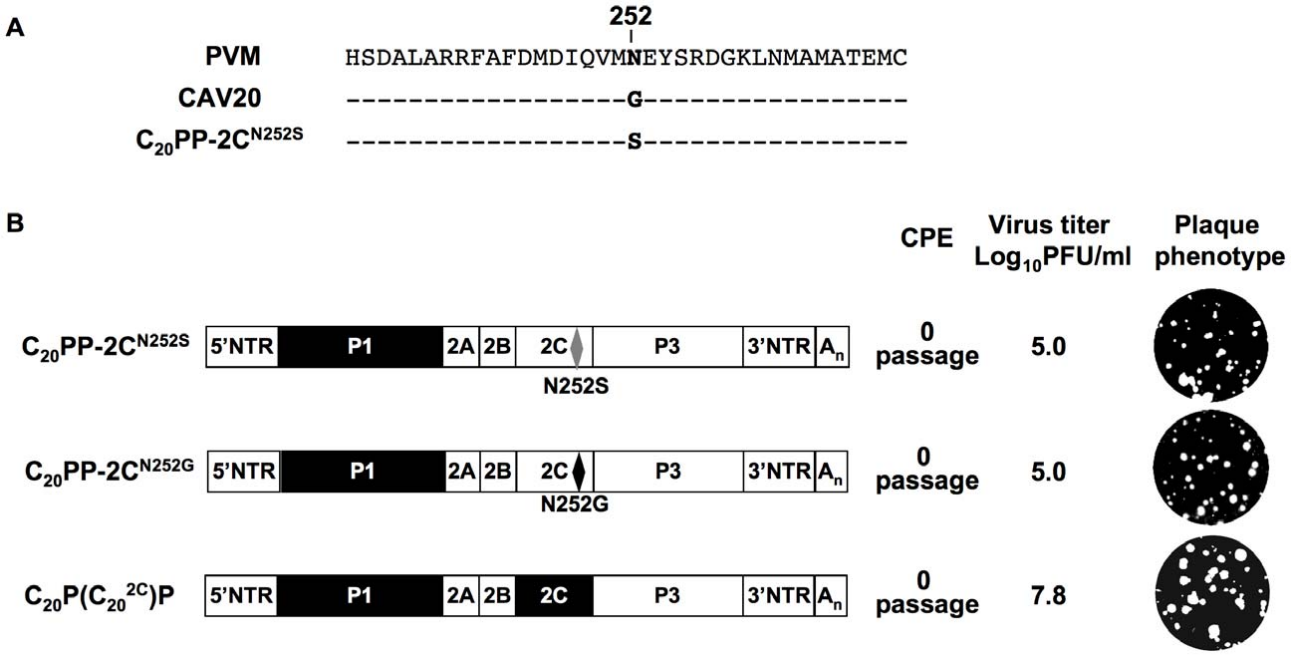
Rescue of the C_20_PP virus by a CAV20-like amino acid or CAV20 2C^ATPase^. (**A**) Comparison of the amino acid sequences of 2C^ATPase^ flanking residue 252 of PVM, CAV20 and C_20_PP-2C^N252S^. (**B**) The genomic structures of the C_20_PP derivatives. Note that the 2C^N252G^ change is different from what was observed in the encapsidation adapted virus (2C^N252S^). N252S mutation in C_20_PP-2C^N252S^ is shown by grey diamond whereas the N252G mutation is a black diamond. For nomenclatures see the legend to Figure 2A. The PVM sequences are shown with open boxes and the CAV sequences by black boxes. RNA transcripts were transfected into HeLa H1 cells and at the time of CPE the virus titers were determined by a plaque assay (Materials and Methods).

It should be noted that the mutation in 2C^ATPase^, which rescues in part the encapsidation of C_20_PP-2C^N252S^, is an N/S change in PV 2C^ATPase^ at position 252 (Figure 4A). Amino acid sequence alignment demonstrated that CAV20 has a Gly at this position (Figure 4A), an observation indicating that a CAV-20 like, uncharged residue might be favorable at this position. Thus, since C_20_PP-2C^N252S^ expressed a severe encapsidation phenotype, it was of interest to determine whether a C_20_PP-2C^N252G^ mutant would yield a chimera equal or superior in encapsidation to C_20_PP-2C^N252S^. Similar to C_20_PP-2C^N252S^, the N252G mutation in the PV 2C^ATPase^ only partially rescued the encapsidation phenotype of C_20_PP (Figure 4B). The observed N/S mutation in a naturally selected escape mutant can be explained by the reasoning that the CAV20-like N/G change in codon N252 would have required two nucleotide changes (AAT/GGT) while the N/S mutation entails only a single nucleotide change (AAT/AGT). Overall, the CAV20 like single amino acid change at 252 of PV 2C^ATPase^ was favorable but not sufficient to fully rescue the VP3-related function of the PV 2C^ATPase^ protein in encapsidation.

Based on this observation, we reasoned that the replacement of the entire 2C^ATPase^ coding region in C_20_PP with its CAV20 counterpart should yield a chimera whose protein/protein interaction required for encapsidation would be sufficient and, thus, yield a virus with proliferation phenotypes similar to that of wt CAV20. Therefore, we generated a chimera designated as C_20_P(C_20_^2C^)P that showed CPE after transfection with a virus titer comparable to that of wt CAV20 (compare Figures 2A and 4B). These results provide further support for the hypothesis that the 2C^ATPase^ protein is a partner required for encapsidation. It should be noted that the chimera C_20_P(C_20_^2C^)P had a growth phenotype more similar to that of the wt CAV20 virus than the chimera C_20_PP-2C^N252S^ (compare Figures 2A and 4B), an observation indicating that sequences besides residue 252 in 2C^ATPase^ are also important for function during viral encapsidation.

### The encapsidation of other C-CAV chimeras requires capsid protein(s) and 2C^ATPase^ of the same origin

**Figure 5:**
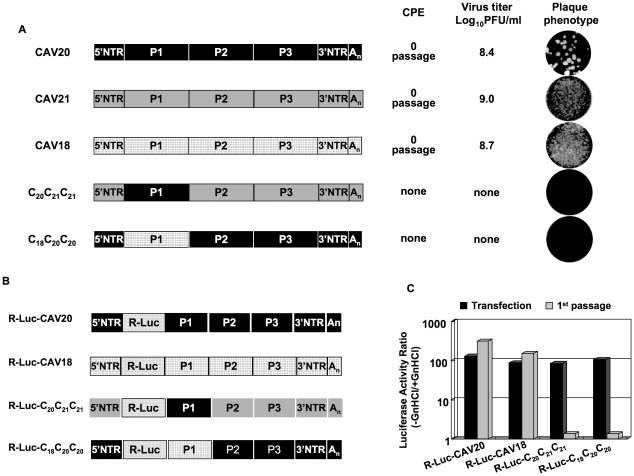
Chimeras of C-CAVs, C_20_C_21_C_21_ and C_18_C_20_C_20_, are defective in encapsidation. (**A**) Growth phenotypes of parental and of chimeric viruses. The genomic structures of the parental and chimeric viruses are shown on the left. CAV20 sequences are shown by black boxes, CAV21 by gray boxes and CAV18 by hatched boxes. The nomenclature for the chimeric viruses is given in the legend to Figure 2. RNA transcripts were transfected into HeLa H1 cells and the viruses obtained at CPE, if any, were titered by plaque assay (Materials and Methods). (**B**) The genomic structures of the parental and chimeric luciferase reporter viruses. (**C**) Comparison of RNA replication and encapsidation of the parental C-CAVs and of chimeric C-CAVs reporter viruses. To determine the level of RNA replication (black bar), RNA transcripts were transfected into HeLa H1 cells both in the absence and presence of 2 mM GnHCl. To determine encapsidation (grey bar), the transfected cell lysates were passaged into fresh HeLa H1 cells in the absence and presence of GnHCl. The luciferase activity in the absence (−GnHCl) and presence of 2 mM GnHCl (+GnHCl) was measured and the luciferase activity ratio (–GnHCl/+GnHCl) was calculated to quantify the extent of replication (Materials and Methods). The luciferase data are averages of at least three independent experiments.

The observation that CAV20 VP3 needs its own 2C^ATPase^ to fully rescue the defect in encapsidation suggests that the cooperation or interaction between 2C^ATPase^ and capsid might be specific and generally true for C-HEVs. To test this hypothesis we extended our analyses to CAV18 and CAV21, two viruses that are phylogenetically related to CAV20 and PV [48]. Chimeras C_20_C_21_C_21_ and C_18_C_20_C_20_ (Figure 5A) displayed non-viable phenotypes as judged by the lack of virus in plaque assays even after 8 blind passages on fresh HeLa H1 monolayers (Figure 5A). In order to test whether the lethal phenotypes of the two chimeric viruses were due to the same encapsidation defect as that of C_20_PP, we constructed reporter viruses of CAV18 and of the chimeras C_20_C_21_C_21_ and C_18_C_20_C_20_ (Figure 5B) just as that of CAV20 (Figure 2C). After transfection of RNA transcripts into HeLa H1 cells, R-Luc activity at 16-hr post transfection showed that the parental and chimeric viruses replicated their genomes with nearly wt efficiency (Figure 5C). However, after the first passage on fresh HeLa H1 cells only wt CAV20 and wt CAV18 reporter viruses yielded normal luciferase signals (Figure 5C). The chimeric genomes R-Luc-C_20_C_21_C_21_ and R-Luc-C_18_C_20_C_20_ could not produce an infection, a result demonstrating an encapsidation defect.

**Figure 6:**
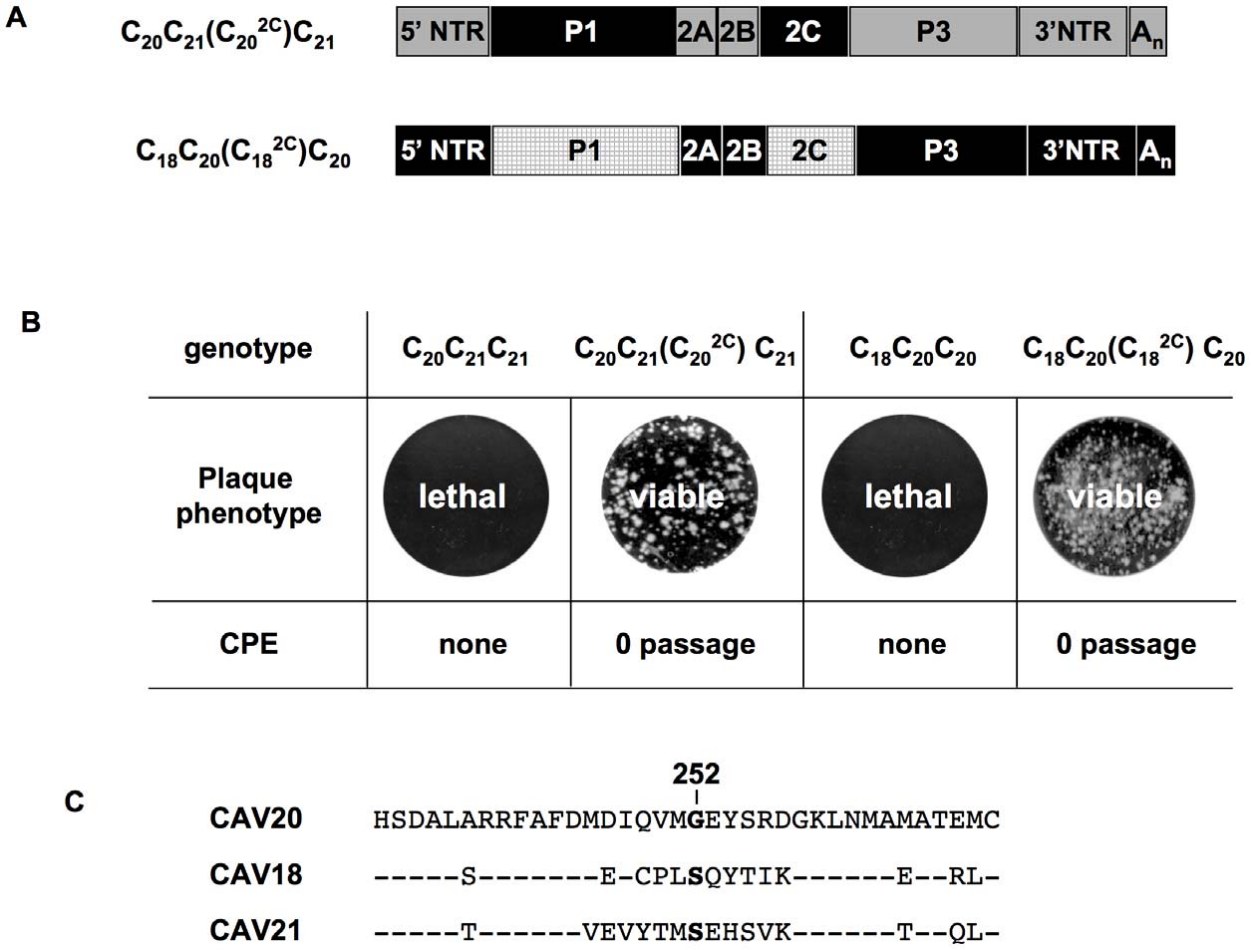
Rescue of lethal C-CAV chimeras by 2C^ATPase^ of the same origin as P1. (**A**) Genomic structures of the chimeric viruses containing both the P1 and 2C^ATPase^ regions from the same origin. For the genomic structures of the chimeric viruses with only P1 exchanges see Figure 4A. CAV20 sequences are shown with a black box, CAV21 sequences with a gray box and CAV18 sequences with a hatched box. For the nomenclature of the chimeric viruses see the legend to Figure 2. (**B**) Comparison of growth phenotypes of the chimeric viruses with just P1 or with P1+2C^ATPase^ exchanges. RNA transcripts were transfected into HeLa H1 cells and the plaque phenotype of the virus, if any, was tested at the time of CPE (Materials and Methods). (**C**) Comparison of the amino acid sequences flanking residue 252 of 2C^ATPase^ among CAV20, CAV18 and CAV21.

To test whether the lethal proliferation phenotypes of the two chimeras (C_20_C_21_C_21_ and C_18_C_20_C_20_) are related to an incompatibility between the capsid and the 2C^ATPase^ proteins derived from different parental viruses, we constructed new chimeras [C_20_C_21_(C_20_^2C^)C_21_, C_18_C_20_(C_18_^2C^)C_20_] in which the capsid and 2C^ATPase^ proteins were derived from the same origin (Figure 6A). The resulting chimeras all showed CPE after transfection (Figure 6B) and the virus titers were comparable to that of the parental viruses (data not shown). The finding that the lethal growth phenotypes of the chimeras were fully rescued when the capsid and 2C^ATPase^ were derived from the same origin serves as further support of our hypothesis that 2C^ATPase^ and capsid proteins communicate with each other during the process of encapsidation. So far, we have not been able to determine, by continued passage, the necessary amino acid changes in the 2C^ATPase^ protein of CAV20 and CAV21 to allow encapsidation of the C_20_C_21_C_21_ and C_18_C_20_C_20_ chimeras. This observation might be explained by the fact that there are many amino acid differences either between CAV20 and CAV21 or between CAV20 and CAV18 flanking residue 252 of the 2C^ATPase^ protein (Figure 6C). This may make it difficult for the two chimeras (C_20_C_21_C_21_ and C_18_C_20_C_20_) to generate escape mutants simply by natural selection during passages.

### A direct interaction between proteins 2C^ATPase^ and VP3 is revealed by biochemical assays

**Figure 7:**
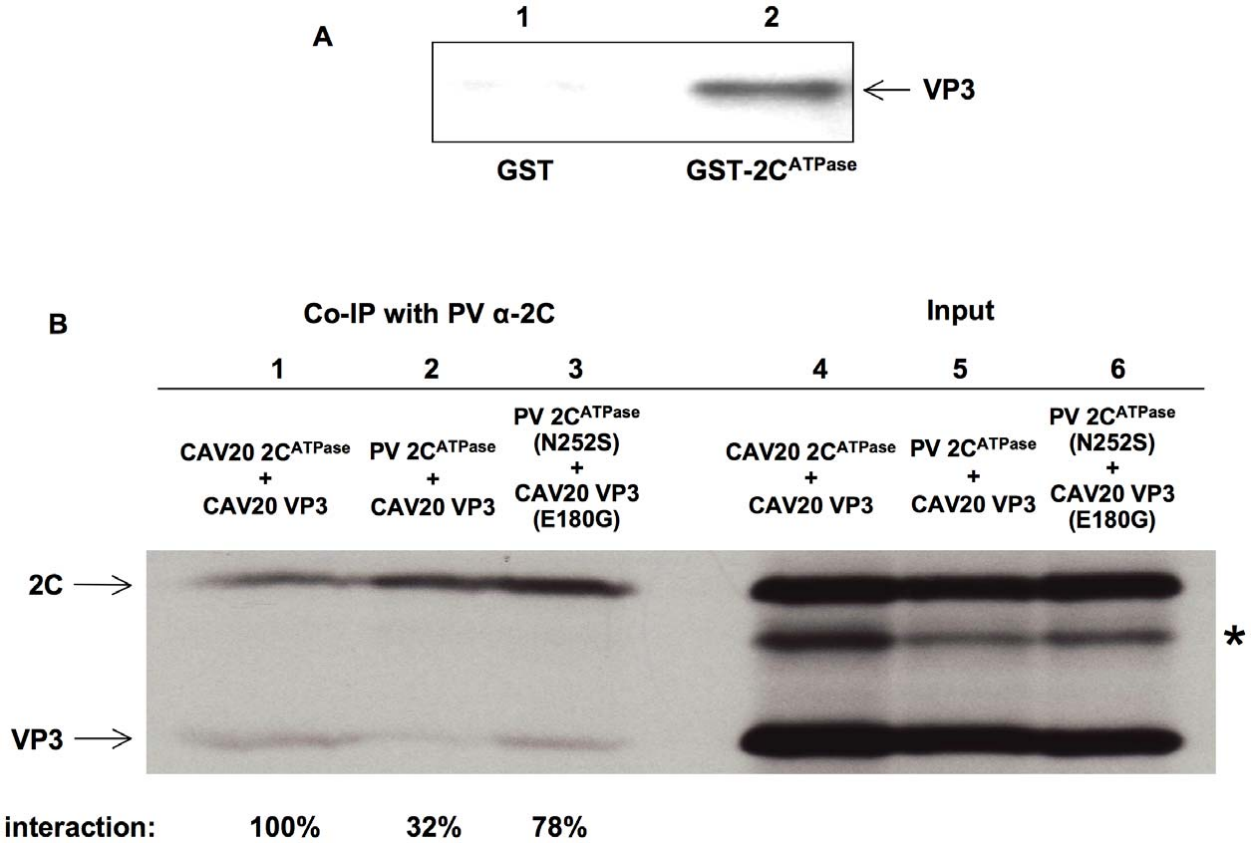
Direct interaction between VP3 and 2C^ATPase^ demonstrated by biochemical assays. (**A**) GST pull down assay. PV GST-2C^ATPase^, PV VP3-His and GST were purified as described in Materials and Methods. Lane 1: GST was used to pull down PV VP3-His; Lane 2: PV GST-2C^ATPase^ was used to pull down PV VP3-His. (**B**) Co-immunoprecipitation (Co-IP) assays. RNA transcripts containing 2C and VP3 coding sequences of PV or CAV20, were co-translated in HeLa cell extracts in the presence of ^35^S-methionine. Lane 4: input CAV20 2C^ATPase^ & CAV20 VP3; Lane 5: input PV 2C^ATPase^ & CAV20 VP3; Lane 6: input PV 2C^ATPase^ (N252S) & CAV20 VP3 (E180G). The positions of 2C^ATPase^ and VP3 are indicated. The position of incomplete translation products of RNA transcripts of 2C coding sequence is indicated by asterisk. Using polyclonal antibody against PV 2C^ATPase^, Co-IP assays were done as described in Materials & Methods. The co-immunoprecipitated protein products from co-translation reactions shown in lanes 4, 5 and 6 were loaded in Lanes 1, 2, 3, respectively, analyzed by SDS-PAGE, and detected by autoradiography. The extent of interactions was quantified and calculated as percentages of the levels observed in the CAV 2C^ATPase^ and CAV VP3 Co-IP reaction (shown below).

The genetic evidence described above strongly suggested a direct interaction between the capsid proteins and 2C^ATPase^, which is required for encapsidation. To confirm this interaction, we carried out a GST-pull down assay with purified PV proteins GST-2C^ATPase^ and His-VP3 (Figure 7A, lane 2). The same assay was performed with purified GST protein as a control (Figure 7A, lane 1). Our results clearly showed that PV GST-2C^ATPase^ interacts directly with the PV His-tagged VP3 protein. To provide further proof that direct interaction between VP3 and 2C^ATPas^ is required for the encapsidation process, co-immunoprecipitation (Co-IP) assays were performed with VP3 and 2C^ATPase^ proteins in three different combinations: CAV20 VP3 & CAV20 2C^ATPase^, CAV20 VP3 & PV 2C^ATPase^, CAV20 VP3 (E180G) & PV 2C^ATPase^ (N252S), which correspond to those observed in wt CAV20, nonviable chimera C_20_PP, and rescued C_20_PP-DM, respectively. *In vitro* transcribed RNA transcripts of 2C and VP3 coding sequences in different combinations were co-translated in HeLa cell extracts (Figure 7B, lanes 4–6) [11], [55]. Using PV 2C polyclonal antibody, which recognizes PV 2C^ATPase^ and CAV20 2C^ATPase^ with the same efficiency (data not shown) [56], CAV20 VP3 was co-immunoprecipitated readily by CAV20 2C^ATPase^ (100%, Figure 7B, lane 1) but only weakly by PV 2C^ATPase^ (32%, Figure 7B, lane 2). The extent of interaction between 2C^ATPase^ and VP3 was quantified using a PhosphorImager and is expressed as percentage of the level observed in the CAV20 2C^ATPase^ and CAV20 VP3 Co-IP reaction. These results indicated a strong, direct interaction between 2C^ATPase^ and VP3 of the same origin (CAV20) but not between PV 2C^ATPase^ and CAV20 VP3, a combination that yielded the nonviable C_20_PP chimera. In contrast, the interaction between PV 2C^ATPase^ (N252S) and CAV20 VP3 (E180G) proteins was restored (78%, Figure 7B, lane 3) when the two mutations were incorporated into PV 2C^ATPase^ and CAV20 VP3, respectively, This observation, which correlates with the rescue of the nonviable phenotype of C_20_PP by the two mutations, strongly support the notion that sufficient protein-protein interaction between 2C^ATPase^ and VP3 is essential for the encapsidation process. The same Co-IP assays were also performed with α-actin antibody and empty resin as controls to ensure that the interactions were not due to non-specific binding of the proteins to the antibody or to the resin (data not shown). It should be noted that there was an extra protein band shown below the band of 2C^ATPase^ in each of the input lanes (Figure 7B, lane 4–6, indicated by asterisk), which was possibly generated from the internal initiation or premature termination of the translation of RNA transcripts of 2C coding sequences. Apparently these incomplete translation products could not be recognized by the 2C antibody since protein bands disappeared after Co-IP (Figure 7B, lane 1–3). These results confirm the specificity of the Co-IP assay in which the detection of the VP3 protein was due to its co-immunoprecipatation with the 2C^ATPase^ protein, recognized by anti-2C^ATPase^ antibody.

The data support our hypothesis that 2C^ATPase^ is required for viral encapsidation through a direct interaction with capsid protein VP3 and also confirm that 2C^ATPase^ interacts with VP3 through protein–protein rather than RNA-protein interaction. Given that 2C^ATPase^ and capsid proteins are colocalized on the surface of membranous vesicles in the RNA replication complex [46], [47], it is likely that 2C^ATPase^ interacts with VP3 either in the form of the mature protein or in the context of one of the VP3-containing capsid precursors (5S, 14S, 75S and 150S).

## Discussion

The mechanism of picornavirus genome encapsidation has been a conundrum for many years. In previous studies on poliovirus morphogenesis, mostly *trans* encapsidation experiments were performed to determine the specificity of PV morphogenesis. In *trans* encapsidation experiments, the capsid proteins are offered from a different molecular entity to the parental genome either by coinfecting picornaviruses [14], by the vaccinia system [22], [23] or by expression from co-transfected cDNAs [64]. However, differences in the experimental design also affect the outcome of the experiments. Jia et al., [64] have reported *trans*-encapsidation of PV replicons into capsids of coxsackie B3, human rhinovirus 14, and coxsackie B24 viruses. These data, however, are in contrast to those of Porter et al., [23] and Barclay et al., [14], who failed to *trans* encapsidate the reporter PV replicon by super-infecting with CAV21.

In the current study, we have used a novel system to study encapsidation of closely related enteroviruses, the C-cluster enteroviruses (C-HEVs). They consist of two classes of virus serotypes: PV and C-CAVs. We have made use of differences between these viruses to investigate the effect of capsid exchanges on C-HEV morphogenesis. By studying a variety of C-HEV chimeric viruses in which the capsid P1 precursors were exchanged, we now present direct evidence for the involvement of 2C^ATPase^ in enterovirus morphogenesis via direct interaction with capsid protein VP3. Different from *trans* encapsidation assays, in which the capsid proteins are offered from a different molecular entity to the parental genome, our experiments were designed to measure *cis* encapsidation of genomes, in which the capsid is provided (generated) by the chimeric genome itself. It appears that in these chimeras the non-structural proteins are more discriminatory to capsid proteins because they are required to proteolytically process the heterologous capsid precursor. In addition, the quality and quantity of the heterologous capsid proteins produced might not be ideal for the chimeric genome. As we have shown here, favorable conditions for encapsidation are rarely met in the chimera, even if the viruses are as closely related as PV and CAV20.

In a previous study on genetic recombination between PV and C-CAVs, we observed that capsid chimera C_20_PP initially could not grow [48], an observation indicating that it harbors defect(s) debilitating the viral replication life cycle. Translation in HeLa cell extracts showed, however, that both translation and proteolytic processing of the CAV20 capsid precursor in C_20_PP was unimpaired. This is not surprising since the 3CD^pro^ proteins of poliovirus and CAV20 are closely related in amino acid sequence [48] and because the 3CD^pro^ cleavage sites within the P1 precursor of PVM and CAV20/21 are well conserved (Supplementary Table 1 in Text S1). These two facts reduce the likelihood of a processing defect of the foreign capsid precursor prior to packaging. In contrast, a chimera consisting of the PV capsid and the coxsackie virus B3 (CBV3) nonstructural domains was not only dead but it also revealed a processing defect of the PV capsid precursor [65]. CBV3 is an enterovirus belonging to the B-cluster, and its genetic kinship with PV is much more distant than that between PV and CAV20. Thus, CBV3 3CD^pro^ proteinase was apparently unable to properly cleave the PV capsid precursor. Similar results were obtained with a chimera of PV in which the 3C-coding region was derived from HRV14. The foreign proteinase was not capable of recognizing the PV-specific processing sites within the capsid precursor [57].

The robust RNA replication phenotype of C_20_PP demonstrated here with the use of a new reporter virus construct suggested to us that this chimera might be quasi-infectious with respect to encapsidation. This was proven to be correct since viable viruses were found upon blind passages of C_20_PP but they harbored mutations. The virus isolates from two independent transfections contained a mutation either in 2C^ATPase^ (N252S) or in VP3 (E180G). Either of these single mutations was able to partially rescue the defective encapsidation and growth phenotype of C_20_PP. Introducing both mutations together into the C_20_PP genome fully rescued packaging and resulted in normal production of progeny virus. It is noteworthy that we never found both mutations in a single isolate even after eight passages. Perhaps the mutations conferred to the variants too little of an advantage to be selected under the conditions of the experiments. As discussed earlier, an involvement of essential RNA sequences in the 2C^ATPase^ and VP3 coding sequences during the process of encapsidation can be excluded. A direct interaction between the 2C^ATPase^ and VP3 proteins, suggested by the genetic experiments, was confirmed by biochemical assays using either purified or *in vitro* translated PV and CAV20 proteins. It should be noted that 2C^ATPase^ also functions in the viral life cycle in the form of its precursor 2BC^ATPase^. A requirement of an interaction between 2BC^ATPase^ and VP3 for packaging is, however, unlikely since 2B is less homologous between PV and CAV20 in sequence than 2C^ATPase^ and the exchange of the mature 2C^ATPase^ protein alone is sufficient for full rescue. We are currently investigating whether the interaction between 2C^ATPase^ and VP3 involves the mature VP3 polypeptide or one of the capsid intermediates during viral assembly and/or maturation.

The encapsidation defect of C_20_PP could also be rescued by replacing the entire 2C^ATPase^ coding sequence of PV with that of CAV20. Additional experiments indicated that the lethal growth phenotypes of other CAV/CAV capsid chimeras could also be reversed by replacing their 2C^ATPase^ coding sequence with that of the capsid donor virus. It is noteworthy that these observations are not contradictory to the scenario of another chimera previously described. PC_20_C_20_, which, in contrast to C_20_PP, possesses a chimeric genome encoding PV capsid and CAV20 nonstructural protein sequences, grows as well as wt PV [48]. We have previously proposed that the evolutionary direction is from C-CAV to PV within C-HEVs resulting in a receptor switch from ICAM-1 to CD155 during the speciation [48]. If so, the newly emerged PV capsid may still be compatible with 2C^ATPase^ of the C-CAV ancestors and achieve sufficient interaction required for encapsidation.

Our data also clarify some unanswered questions about previous *trans* encapsidation experiments using poliovirus replicons containing a reporter gene in the capsid-coding region [14], [23]. Those studies showed that CAV21 was not able to encapsidate a PV replicon even though co-infection of cells with CAV21 resulted in high levels of replication of both CAV21 and the PV replicon. The most likely reason for those results is that the CAV21 capsid, similar to CAV20 capsid, fails to properly interact with PV 2C^ATPase^. From their studies with hydantoin, Vance et al., have suggested that 2C^ATPase^ might have a role in encapsidation by an association of the progeny RNA with the capsid [32]. In other experiments with the same drug, however, Oh et al., [66] have recently proposed that hydantoin inhibits the release of the progeny RNA from the replication complex prior to encapsidation. Whether the interaction between 2C^ATPase^ and VP3, as we have observed in our studies, is required during or just before the union of the RNA with capsid proteins remains to be determined.

**Figure 8:**
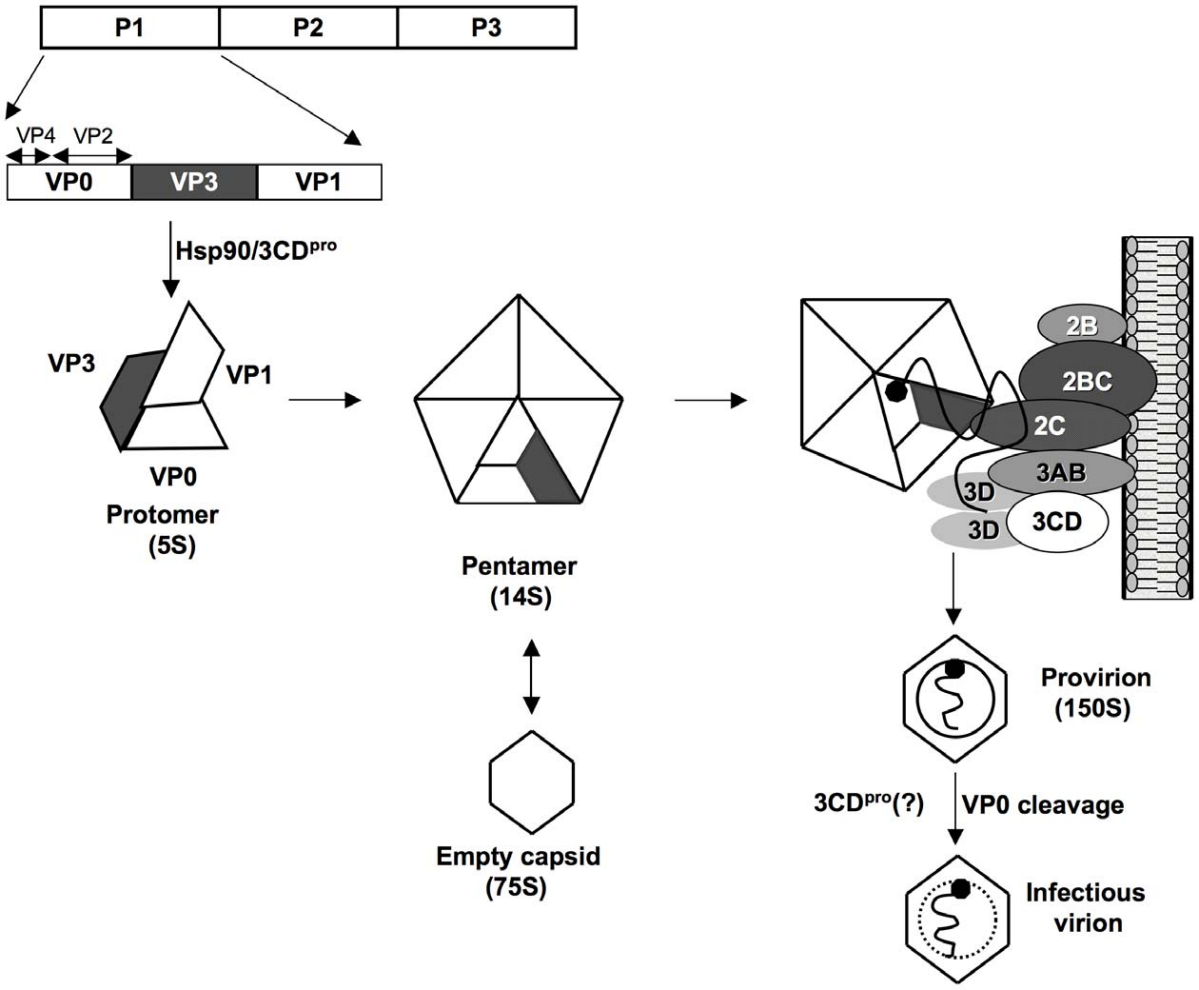
Model for C-HEVs morphogenesis involving the interaction between 2C^ATPase^ and capsid protein VP3. Following its release from the polyprotein by 2A^pro^, P1 is first properly folded by chaperone Hsp90 before it becomes a substrate for 3CD^pro^ [4], [5]. Mature VP0, VP1 and VP3 assemble into a protomer, 5 of which subsequently form a pentamer. Some pentamers generate 75S empty capsids. Progeny viral RNA, released from the replication complexes, is associated with pentamers [9] through their VP3 domains that interact with 2C^ATPase^ on the surface of membranous replication complexes. 12 pentamers assemble to enclose the viral RNA forming a provirion. Maturation cleavage of VP0 to VP2 and VP4 yields an infectious virion. The role of 3CD^pro^ at this step is not yet understood [30].

An intriguing phenomenon of poliovirus encapsidation is that only newly replicated RNA molecules are incorporated into virions [11], [12], an observation reported also for some other RNA viruses such as flock house virus [67], [68], [69], and brome mosaic virus [70]. Coupling encapsidation specifically with replication offers an efficient mechanism of discriminating against cellular RNAs or viral mRNA. Since genome replication is coupled with translation [71], [72] the link to encapsidation “can impose a form of late proofreading” for the progeny virus [12]. In Figure 8, we present a model of morphogenesis that is based, admittedly, on much speculation. We currently propose that in the context of the membrane-associated replication complex 2C^ATPase^ will directly interact with a 14S pentamer via VP3. The pentamer will then bind the newly emerging, VPg-linked genomic RNA [9] while the assembly of the virion proceeds in close contact with the membranous environment. This model offers a new mechanism for the specificity of enterovirus encapsidation: it is dependent on protein/protein interactions at the site of the active replication complex.

It is likely that our model will be applicable to most, if not all, picornaviruses as well as to other families of plus strand RNA viruses. For example, the requirement for an interaction between a capsid protein and nonstructural proteins for encapsidation has also been observed for members of the *Flaviviridae*. Murray et al., reported that several assembly-deficient core mutants of HCV genotype 2a (*Hepacivirus* genus) could be rescued by compensatory mutations in p7 or NS2 [73]. In addition, it was shown that HCV core and NS5A colocalize on the surface of lipid droplets, a process required for particle assembly [74]. Furthermore, recent reports indicate the importance of nonstructural proteins in the maturation of Kunjin virus and Yellow fever virus (*Flavivirus* genus) [75], [76] and of bovine diarrhea virus (*Pestivirus* genus) [77].

We have noted before that Aichi virus, a member of the *Kobuvirus* genus in the *Picornaviridae*, requires a 5′-terminal RNA element for encapsidation [29]. This signal by itself, however, is not sufficient to confer encapsidation specificity since it can be replaced by a similar stem loop from hepatitis A virus, a member of the genus *Hepatovirus* of *Picornaviridae*, and the resulting chimera expresses a severe proliferation phenotype [27]. On the other hand different genera of *Picornaviridae* may have evolved different strategies of encapsidation. This is not entirely unlikely considering the fundamentally different strategies that different genera of *Flaviviridae* are using to control genome translation (cap-dependent vs. IRES-dependent initiation of translation [78].

Our model does not explain, however, how VPg-linked minus-stranded poliovirus RNA is discriminated against in encapsidation. Previous studies have reported that several picornaviral 2C^ATPase^ proteins bind specifically to the 3′-end of minus strand RNA *in vitro* [79], [80]. However, the importance of this interaction, if any, for encapsidation is unlikely. Normally, plus strands are produced in great excess over minus strands [81], a phenomenon thought to lead to the depletion of free minus strands by forming the replicative form (RF) or replication intermediates (RI) [18]. If the balance between the plus and the minus strands is disturbed, free minus strands may emerge from the replication complex and they may then be encapsidated. Indeed, encapsidation of both plus and minus strand genomic RNAs was observed in non-cytopathogenic CBV3 that were isolated from persistently infected murine hearts and cardiac myocyte cultures [82]. It is possible that the non-cytopathogenic CBV3 produces minus stranded RNA in excess such that it will emerge from replication complexes where capsid precursors are waiting to encapsidate them. Whether the “cap” of a positively charged VPg on both plus or minus strands plays a role in this process remains to be seen.

## Supporting Information

Text S1 Supplementary Tables 1–5
(0.09 MB DOC)
