# Supplementary material for: Direct Interaction between Two Viral Proteins, the Nonstructural Protein 2CATPase and the Capsid Protein VP3, Is Required for Enterovirus Morphogenesis
Source: PLoS Pathog. 2010 Aug 26;6(8):e1001066. doi: 10.1371/journal.ppat.1001066 (PMC2928791; doi:10.1371/journal.ppat.1001066)
Supplement: Text S1 — Supplementary Tables 1–5 (0.09 MB DOC) [file ppat.1001066.s001.doc]

**Supplementary Table 1: Sequences flanking the predicted cleavage sites in C-cluster enteroviruses.**

|  | ***VP4/VP2 | **VP2/VP3 | **VP3/VP1 | *VP1/2A | **2A/2B |
| --- | --- | --- | --- | --- | --- |
| PVM | SPML**N**/**S**PNIE | LPRL**Q/G**LPVM | KALA**Q/G**LGQM | DLTT**Y/G**FGHQ | EAME**Q/G**ITNY |
| CAV20 | APAL**N/S**PNVE | VPST**Q/G**LPVM | TAMP**Q/G**IEDL | DLTT**Y/G**FGHQ | EAME**Q/G**ISNY |
| CAV18 | APAL**N/S**PNIE | I PAT**Q/G**LPVM | SAMP**Q/G**LEDL | NLTT**Y/G**FGHQ | EAME**Q/G**ITSY |
| CAV21 | APAL**N/S**PNVE | VPVH**Q/G**LPTM | IGRT**Q/G**IEDL | S I TT**F/G**FGHQ | EAME**Q/G**ITSY |

|  | **2B/2C | **2C/3A | **3A/3B | **3B/3C | **3C/3D |
| --- | --- | --- | --- | --- | --- |
| PVM | YVTK**Q/G**DSWL | EALF**Q/G**PLQY | FAGH**Q/G**AYTG | TAKV**Q/G**PGFD | FTQS**Q/G**EIQW |
| CAV20 | TVIR**Q/G**DSWL | EALF**Q/G**PLQY | FAGH**Q/G**AYTG | TAKV**Q/G**PGFD | AVTE**Q/G**YLNL |
| CAV18 | FVMK**Q/S**DSWM | EALF**Q/G**PISY | FAGQ**Q/G**AYTG | TAKV**Q/G**PGFD | FTQS**Q/G**EIQW |
| CAV21 | IVMR**Q/G**DGWM | EALF**Q/G**PLRY | FAGQ**Q/G**AYTG | VAKV**Q/G**PGFD | FTQN**Q/G**EIQW |

*VP1/2A cleavage (Y/G or F/G) is catalyzed by 2A^pro^.

**Other cleavages except VP4/VP2 cleavage (Q/G) is catalyzed by 3C^pro^ or its precursor 3CD^pro^

***VP4/VP2 cleavage (N/S) is catalyzed by unknown proteinase.

**Supplementary Table 2:** **Oligonucleotides and templates used for constructing chimeric genomes**

|  | F1 | F2 | F3 |
| --- | --- | --- | --- |
| C_20_C_21_C_21_ | Sense 5’-CTCCC*CACCGGCG*ACGGTGGCC-3’  Antisense 5’-TGCGCACCCATTTGCACTGTTTATTG-3’  Template pGEMCAV21 | 5’-AACAGTGCAAATGGGTGCGCAAGTTTCAT-3’  5’-GACCAAACCCATAGGTAGTTAAATCCTTG-3’  pT7CAV20 | 5’-TTAACTACCTATGGGTTTGGTCATCAGAA-3’  5’-TTTGTGGAA*AGGCCT*TGCAATTAATGG-3’  pGEMCAV21 |
| C_18_C_20_C_20_ | Sense 5’-GTCCCT*CACCGGTG*ACGGTGGTCC-3’  Antisense 5’-ACCCATTATGATGCAACTGTTTGATT-3’  Template pT7CAV20 | 5’-TTGCATCATAATGGGTGCACAAGTTTCTT-3’  5’-GACCAAATCCATAAGTAGTTAAGTTCTTG-3’  pT7CAV18 | 5’-ACTACTTATGGATTTGGTCACCAAATAAG-3’  5’-GGAAGGT*GGGCCC*TACAAATGACACAG-3’  pT7CAV20 |
| C_20_P(C_20_^2C^)P | Sense 5’-ATAG*CGTACG*CGCCCCCG-3’  Antisense 5’-AACCAACTATCACCTTGCTTGATGAC-3’  Template pT7C_20_PP | 5’-CTTATGTCATCAAGGTGATAGTTGGTTAA-3’  5’-CTGGAGTGGTCCCTGGAACAAGGCCTCC-3’  pT7CAV20 | 5’-GAGGCCTTGTTCCAGGGACCACTCCAG-3’  5’-TGTT*CCTAGG*ATCTTTAGTCC-3’  pT7C_20_PP |
| C_20_C_21_(C_20_^2C^)C_21_ | Sense 5’-CAACGAT*GGTTACC*AGCACTATC-3’  Antisense 5’-CTATCACCTTGGCGCATCACATAAG-3’  Template pGEMCAV21 | 5’-GATGCGCCAAGGTGATAGTTGGTTAAA-3’  5’-AGTGGTCCCTGGAACAAGGCCTCCATG-3’  pT7CAV20 | 5’-TTGTTCCAGGGACCACTAAGGTATAAA-3’  5’-GGTTTC*TCCTGGA*GCGGCATG-3’  pGEMCAV21 |
| C_18_C_20_(C_18_^2C^)C_20_ | Sense 5’-ATTTGTA*GGGCCC*ACCTTCC-3’  Antisense 5’-TGAATCACTCTGTCTGATAACGTAG-3’  Template pT7CAV20 | 5’-TATCAGACAGAGTGATTCATGGATGAAG-3’  5’-TAGTGGTCCTTGGAACAAAGCTTCCATG-3’  pT7CAV18 | 5’-TTGTTCCAAGGACCACTACAATACAGA-3’  5’-CCTGGGA*GTCGAC*TGCTTGAAG-3  pT7CAV20 |

Enzyme restriction sites used for cloning are shown in italic and underlined.

**Supplementary Table 3: Oligonucleotides and templates used for constructing C_20_PP derivatives**

|  | F1 | F2 |
| --- | --- | --- |
| C_20_PP-VP3^E180G^ | Sense 5’-ATAG*CGTACG*CGCCCCCG-3’  Antisense 5’-TAAAGTCATC**CCC**CACCGTGCGCCTGTATGC-3’  Template pT7C_20_PP | 5’-ATACAGGCGCACGGTG**GGG**GATGACTTTACAGAAG-3’  5’-ATTTT*GGTGACC*AAATCCATACG-3’  pT7C_20_PP |
| C_20_PP-VP3^N252S^ | Sense 5’-AGAAA*CTCGAG*CATACTATTAAC-3’  Antisense 5’-CATTCAGGTCATG**AGT**GAGTATTCTAGAGATG-3’  Template pT7C_20_PP | 5’-CCCATCTCTAGAATACTC**ACT**CATGACCTGAATGTC-3’  5’-TGTT*CCTAGG*ATCTTTAGTCC-3’  pT7C_20_PP |
| C_20_PP-2C^N252G^ | Sense 5’-AGAAA*CTCGAG*CATACTATTAAC-3’  Antisense 5’-CATTCAGGTCATG**GGT**GAGTATTCTAGAGATG-3’  Template pT7C_20_PP | 5’-CCCATCTCTAGAATACTC**ACC**CATGACCTGAATGTC-3’  5’-TGTT*CCTAGG*ATCTTTAGTCC-3’  pT7C_20_PP |

Enzyme restriction sites used for cloning are shown in italic and underlined.

Nucleotide changes introduced in cloning are shown in bold.

**Supplementary Table 4: Oligonucleotides and templates used for constructing Renilla Luciferase replicons**

|  | F1 | F2 | F3 |
| --- | --- | --- | --- |
| Rlu-CAV20 | Sense 5’-GTCCCT*CACCGGCG*ACGGTGG-3’  Antisense5’-TGCGCACCCATTATGATGCAACTG-3’  Template pT7CAV20 | 5’-AATGGGTGCGCAAGAAACTTCGAAAGTTTATG-3’  5’-GCGCACCATAGGTAGTTAATTGTTCATTTTG-3  pT7R-Luc-PVM | 5’-AACTACCTATGGTGCGCAAGTTTCATC-3’  5’-GCGGCGTT*GGTACC*ACTGTC-3’  pT7CAV20 |
| Rlu-CAV21 | Sense 5’-GCACTCCC*CACCGGCG*ACGGTG-3’  Antisense 5’-GAAGTTTCCTGAGCACCCATTTGC-3’  Template pGEMCAV21 | 5’-TGGGTGCTCAGGAAACTTCGAAAGTTTATG-3’  5’-CCCCAAAAGTGGTAATTTGTTCATTTTTGAG-3’  pT7R-Luc-PVM | 5’-AATTACCACTTTTGGGGCTCAAGTTTC-3’  5’-GGTTTC*TCCTGGA*GCGGCATG-3’  pGEMCAV21 |
| Rlu-C_20_C_21_C_21_ | Sense 5’-GCACTCCC*CACCGGCG*ACGGTGG-3’  Antisense 5’-TTCGAAGTTTCTTGAGCCCCCAT-3’  Template pGEMCAV21 | 5’-GGGCTCAAGAAACTTCGAAAGTTTATGATC-3’  5’-AAAAGTGGTAATTTGTTCATTTTTGAGAAC-3’  pT7R-Luc-PVM | 5’-ATTACCACTTTTGGTGCGCAAGTTTC-3’  5’-GCGGCGTT*GCTAGC*ACTGTC-3’  pT7CAV20 |
| Rlu-C_18_C_20_C_20_ | Sense 5’-CGTCCCT*CACCGGTG*ACGGTG-3’  Antisense 5’-TTTCGAAGTTTCTTGCGCACCC-3’  Template pT7CAV20 | 5’-GGGTGCGCAAGAAACTTCGAAAGTTTATG-3’  5’-GTGCACCATAGGTAGTTAATTGTTCATTTTTG-3’  pT7R-Luc-PVM | 5’-TTAACTACCTATGGTGCACAAGTTTCTTC-3’  5’-AGGAAAG*CCGCGG*GTCTGATG-3’  pT7CAV18 |

Enzyme restriction sites used for cloning are shown in italic and underlined.

**Supplementary Table 5: cloning vector, restriction sites for construction and linearization of constructed genomes**

|  | Cloning vector | Cloning sites | Linearization site |  | Cloning vector | Cloning sites | Linearization site |
| --- | --- | --- | --- | --- | --- | --- | --- |
| C_20_C_21_C_21_ | pGEMCAV21 | SgrA I (337)  Stu I (4953) | Sal I (7471) | R-Luc-PVM | pT7PVM | Sac I (761) | Pvu I (9127) |
| C_18_C_20_C_20_ | pT7CAV20 | SgrA I (339),  Apa I (3610) | Eag I (7496) | R-Luc-CAV20 | pT7CAV20 | SgrA I (339)  Nhe I (853**)** | Eag I (8426) |
| C_20_P(C_20_^2C^)P | C_20_PP | Bswi I (2190)  Avr II (7235) | SspI (7750) | R-Luc-CAV21 | pGEMCAV21 | SgrA I (337)  BamH I (779) | Sal I (8425) |
| C_20_C_21_(C_20_^2C^)C_21_ | C_20_C_21_C_21_ | BstE II (3892)  Pfo I (5530) | Sal I (7471) | R-Luc-C_20_PP | R-Luc-PVM | Sac I (761)  Stu I (4804) | SspI (8686) |
| C_18_C_20_(C_18_^2C^)C_20_ | C_18_C_20_C_20_ | Apa I (3634),  Sal I (5212) | Eag I (7496) | R-Luc-C_20_C_21_C_21_ | C_20_C_21_C_21_ | SgrA I (337)  Nhe I (824) | Sal I (8425) |
| C_20_PP-VP3^E180G^ | C_20_PP | Bswi I (2190)  BstE II (3892) | SspI (7750) | R-Luc-C_18_C_20_C_20_ | C_18_C_20_C_20_ | SgrA I (339)  Sac II (2041) | Eag I (8503) |
| C_20_PP-2C^N252S^ | C_20_PP | Xho I (4463)  Avr II (7235) | SspI (7750) | R-Luc-C_20_PP  -VP3^E180G^ | C_20_PP-VP3^E180G^ | Esp I (5399)  Sac I (6117) | SspI (8686) |
| C_20_PP-DM | C_20_PP-2C^N252S^ | Bswi I (2190)  BstE II (3892) | SspI (7750) | R-Luc-C_20_PP  -2C^N252S^ | C_20_PP-2C^N252S^ | Esp I (5399)  Sac I (6117) | SspI (8686) |
| C_20_PP-2C^N252G^ | C_20_PP | Xho I (4463)  Avr II (7235) | SspI (7750) | R-Luc-C_20_PP-DM | C_20_PP-DM | Esp I (5399)  Sac I (6117) | SspI (8686) |
